# Medical student teaching in the UK: how well are newly qualified doctors prepared for their role caring for patients with cancer in hospital?

**DOI:** 10.1038/sj.bjc.6603888

**Published:** 2007-07-31

**Authors:** J Cave, K Woolf, J Dacre, H W W Potts, A Jones

**Affiliations:** 1 Academic Centre for Medical Education, Royal Free and University College Medical School, London, UK; 2 Centre for Health Informatics and Medical Education, Royal Free and University College Medical School, London, UK; 3 Department of Oncology, Royal Free and University College Medical School, London, UK

**Keywords:** undergraduate, medical education, oncology, communication skills, palliative care

## Abstract

A number of studies have identified problems with undergraduate oncology teaching. We have investigated how well prepared newly qualified doctors (first foundation year, or FY1 doctors) are for treating patients with cancer. Twenty-five FY1 doctors and 15 senior doctors participated in interviews. We turned the emergent themes into a questionnaire for all 5143 UK FY1 doctors in 2005. The response rate was 43% (2062 responses). Sixty-one percent of FY1 doctors had received oncology teaching at medical school, but 31% recalled seeing fewer than 10 patients with cancer. Forty percent of FY1 doctors felt prepared for looking after patients with cancer. Sixty-five percent felt prepared for diagnosing cancer, 15% felt they knew enough about chemotherapy and radiotherapy, and 11% felt prepared for dealing with oncological emergencies. Respondents believed medical students should learn about symptom control (71%) and communication skills (41%). Respondents who had received oncology teaching were more likely to feel prepared for looking after patients with cancer (OR 1.52; 95% CI 1.14–2.04). Preparedness also correlated with exposure to patients with cancer (OR 1.48; 95% CI 1.22–1.79). We have found worryingly low levels of exposure of medical students to patients with cancer. First foundation year doctors lack knowledge about cancer care and symptom control. Oncologists should maintain involvement in undergraduate teaching, and encourage greater involvement of patients in this teaching.

More than 1 in 3 people will develop cancer during their lifetime (Office for National Statistics, 2004). There is a rising incidence due to ageing of the population, and a rising prevalence due to developments in cancer treatment increasing the number of survivors either still with, or cured from, their cancer. Many of these survivors have physical or psychological sequelae for which they require care. Doctors who are not cancer specialists carry out the majority of the general health care of cancer-diagnosed patients and their families, especially those aspects of health care not directly cancer-related, but upon which the effect of their cancer has to be considered. Examples would include vaccinations, management of community infections, and management of new symptoms for which knowledge of the earlier diagnosis of cancer may be relevant.

Doctors without a specialist interest in cancer have limited opportunities for postgraduate training in oncology, thus their undergraduate training is of great importance. Surveys in Europe (Haagedoorn, 1985; Bender *et al*, 1993; Cellerino *et al*, 1993; Ramakrishnan *et al* 1993), the USA (Bakemeier, 1981), and Australia (Tattersall *et al*, 1993; Barton and Simons, 1999) have all identified problems with undergraduate oncology teaching including inadequate coordination, insufficient resources, and variability in the curriculum (UICC, 1994). Several methods for teaching students about oncology have been evaluated (Gaffan *et al*, 2006), but there is little published evidence to guide the choice of content. An ideal undergraduate oncology curriculum has been developed by consensus in Australia (Australian Cancer Society, 1999; Koczwara and Barton, 2006).

One aim of undergraduate medical training is to prepare students for their first year of work as doctors (The General Medical Council, 1993; Wood, 2003), but evidence shows that this preparation can be poor (Goldacre *et al*, 2003). Newly qualified doctors all look after patients with cancer, and there is concern about how well prepared they are for this role (Barton *et al*, 2003, 2006). Undergraduate oncology teachers need more information about newly qualified doctors' educational needs, so they can ensure doctors leaving medical school are well prepared to care for patients with cancer.

Our aim was to investigate how much oncology teaching UK medical students receive, and how well it prepares them for looking after patients with cancer when they start to work as doctors.

## MATERIALS AND METHODS

This study was conducted in two phases: a qualitative (interview) study and a quantitative (questionnaire) study. The interview results were used to inform the design of the questionnaire.

### Participants

After graduation from UK medical school, newly qualified doctors undergo 12 months of supervised practice, previously called the pre-registration house officer year and now referred to as the first foundation year (FY1). This was a study of FY1 doctors in the UK during 2004–2005.

### Study design

#### Interview study

**Figure 1:**
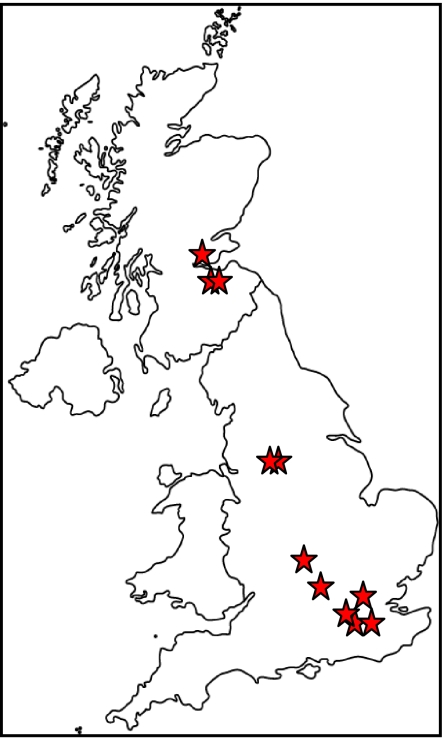
Geographical distribution of PRHOs in the interview study.

Twenty-five FY1 doctors took part in one-to-one semi-structured interviews. Staff from four postgraduate deaneries recruited 23 FY1 doctors. Three more were recruited by their colleagues. The interviewees were purposively sampled so as to be distributed by gender, specialty (medicine or surgery), whether they had a higher degree before medical school entry, and location (Figure 1). Twenty-five out of 26 FY1 doctors agreed to be interviewed.

We also interviewed a group of senior doctors to improve the validity of the qualitative findings by triangulation (Greenhalgh *et al*, 1998; Silverman, 2005). We asked each FY1 interviewee to nominate a senior colleague to take part in the study. Twenty FY1 doctors nominated 22 senior colleagues: 20 consultants, 1 specialist registrar, 1 one primary care physician. They were invited for interview by letter (*n*=9), fax (*n*=5), and/or e-mail (*n*=20) according to telephone advice from their secretaries. Fourteen consultants and one registrar agreed to be interviewed (68%).

One of us (JC) conducted all the interviews. The junior doctors were asked to describe their care of particular patients with cancer, and then to consider which aspects they felt prepared/unprepared for. The senior doctors were asked to describe their juniors' care of patients with cancer. The junior doctor interviews were face to face, while the senior doctor interviews were face to face (*n*=2), by telephone (*n*=11), or by e-mail (*n*=2), according to preference. Interviews lasted 20–60 min.

#### Questionnaire study

The questionnaire was four pages long, printed on one folded sheet of A3, and was accompanied by an introductory letter explaining the purpose of the study (Boynton and Greenhalgh, 2004). The majority of questions invited answers on a five-point Likert scale from ‘strongly agree’ to ‘strongly disagree’. To avoid bias due to the acquiescence effect, some questions were phrased in the positive and some in the negative. There were also three questions inviting free text responses.

The questionnaire comprised four sections: demographics; questions about preparedness for the FY1 year and caring for patients with cancer; questions about medical school and oncology training; and questions about factors that may influence preparedness such as personality traits. The questions were determined from the previous literature and from the themes that arose in the interview study (Sofaer, 2002). Previously validated instruments were used wherever possible.

Previously published studies show that personality traits affect doctors' attitudes to work (McManus *et al*, 2004) and problem-based learning courses may improve preparedness (Jones *et al*, 2002). Personality traits were measured on an abbreviated version of Costa & McCrae's NEO PI five-factor personality inventory (Costa, 1992). Information about course type at each medical school (problem-based learning or not) was obtained from the school prospectuses.

Data from the interviews with junior and senior doctors suggested the following factors that may affect preparedness: maturity; life experiences; meeting patients with cancer; teaching which is relevant to the foundation year; and feeling supported at work. The questionnaire asked about all of these factors. Possession of a prior degree was recorded as a surrogate for maturity. Data from the interviews generated a list of aspects of patient cancer care commonly carried out by FY1 doctors, and the questionnaire asked respondents to rate their preparedness for each of these aspects of care.

The questionnaire was piloted and then distributed nationally using methods agreed with the individual postgraduate deaneries as follows. In four deaneries, we posted the questionnaires to doctors' work addresses. In the remaining 17 deaneries, the postgraduate training centre administrators gave out the questionnaires. One deanery and eight postgraduate centres declined to participate, because they had previously committed to distribute other surveys. All respondents were entered into a prize draw to win an iPOD (Kalantar and Talley, 1999).

### Data analysis

#### Analysis of interview data

KW and JC transcribed the interviews and performed the analysis by reading the transcripts, first individually and then together, and identifying themes using the constant comparative method (Glaser and Strauss, 1967; Greenhalgh *et al*, 2004). Negative cases that ran counter to the themes were used to refine the analysis (Green, 1998). Towards the end of the study, no new themes emerged, suggesting that saturation had been achieved. KW and JC then independently coded the transcripts using Atlas.ti software (Pope *et al*, 2000).

#### Analysis of questionnaire data

Univariate analyses were conducted using *χ*^2^-tests, and multivariate analyses were conducted using multiple regression and factor analysis. The split half method was used to estimate the reliability of the questionnaire (Rust and Golombok, 1989). All statistical analyses were performed using SPSS for Windows version 12. Free text responses were coded using the constant comparative method (see above).

### Ethics

Ethical approval was obtained through the Central Office for Research Ethics Committees (COREC).

## INTERVIEW RESULTS

Five main themes arose from the FY1 interviews: the doctors talked positively about their oncology training. They described how, both as students and since starting work, they had learned about cancer by meeting individual patients and realising that patients are real people rather than collections of signs and symptoms. They reported that although their communication skills training had prepared them well and role models were helpful, they had difficulty with particular areas of communication including answering patients' questions and talking to relatives. They felt more prepared for curative (e.g. surgical) management of cancer than for palliative care. Finally, they felt better prepared when they felt well supported at work.

The findings from the consultant interviews were consistent with those from the FY1 doctor interviews. The consultants also said that meeting patients, greater maturity, and experiencing ‘life events’ helped prepare students for being doctors.

## QUESTIONNAIRE RESULTS

The response rate was 43% (*n*=2062/4784). The denominator excludes FY1 doctors from the deanery that declined participation in the study and FY1 doctors involved in the pilot study (*n*=359). The numerator excludes respondents who graduated before 2003 (UK graduates) and 2001 (non-UK graduates) because they may have been working in more senior posts than FY1 (*n*=98).

The split half reliability of the questionnaire was 0.75 (Spearman–Brown coefficient). Coefficients above 0.7 are acceptable (Rust and Golombok, 1989).

### Undergraduate oncology teaching and opinions on teaching

Sixty-one percent of respondents (*n*=1249; 95% CI 59–63%) answered ‘yes’ to either the question ‘Did you do an oncology attachment at medical school?’ or the question ‘Did you do an oncology special study module?’ Seventy-six percent (*n*=1545; 95% CI 74–77%) had visited a hospice. Thirty-one percent (*n*=631; 95% CI 29–33%) recalled meeting fewer than 10 patients with cancer at medical school, and nine percent (*n*=185; 95% CI 8–10%) recalled fewer than two patients with terminal cancer. Despite this, only 12% agreed with the statement ‘Medical students are kept away from patients with cancer’ (95% CI 10–13%).

Sixty-one percent of respondents (*n*=1252; 95% CI 59–63%) would have preferred more teaching on oncology. More specifically, 74% (*n*=1528; 95% CI 73–76%) would have preferred to have more teaching on radiotherapy and chemotherapy, and 62% (*n*=1267; 95% CI 60–64%) would have preferred more teaching on symptom control (choices not mutually exclusive).

**Figure 2:**
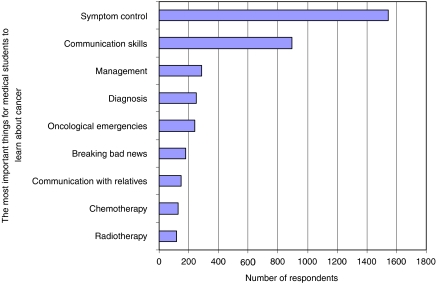
Free text responses to the question ‘What is the most important thing for medical students to learn about cancer?’

Three questions invited free text responses. The first asked how respondents would change teaching at medical school to prepare doctors better for caring for patients with cancer. The most frequent responses were ‘more teaching on symptom control’ (*n*=294, 14%) and ‘more exposure to patients’ (*n*=219, 10%). The second question asked for the most important things to teach about cancer at medical school. The most frequent responses were ‘symptom control’ (*n*=1542, 71%) and ‘communication skills’ (*n*=896, 41%; Figure 2). The third question asked for the least important things to teach about cancer at medical school. The most frequent responses were ‘details of chemotherapy’ (*n*=504, 23%) and ‘details of radiotherapy’ (*n*=276, 13%; ‘*n*’ for these responses varies widely, because many respondents chose not to answer the first and the third questions).

### Preparedness for looking after patients with cancer

Forty percent of respondents agreed or strongly agreed with the statement ‘I felt prepared for looking after patients with cancer’ (*n*=819; 95% CI 38–42%), and 23% disagreed or strongly disagreed (*n*=469; 95% CI 21–25%). Seventeen respondents (0.8%; 95% CI 0.5–1%) said they had no experience of looking after patients with cancer.

**Figure 3:**
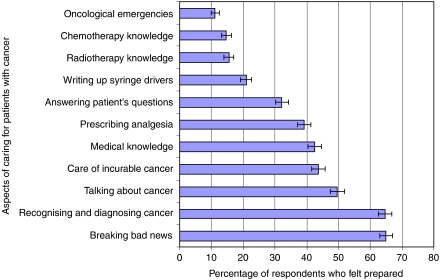
The percentage of respondents who felt prepared for different aspects of cancer care (error bars show 95% CI).

The questionnaire asked about preparedness for specific aspects of care (Figure 3). Preparedness was higher in relation to the process of diagnosis (65% prepared) and breaking bad news (65% prepared) than for dealing with oncological emergencies (11% prepared), knowledge about chemotherapy and radiotherapy (15% prepared), and prescribing drugs for symptom control such as opiates in syringe drivers (21% prepared).

### Variables that correlate with preparedness

A period of oncology teaching appeared to improve preparedness: 44% of those who had done an oncology attachment felt prepared for looking after patients with cancer, compared with 34% of those who had not (*χ*^2^=17.9; df=1; *P*<0.001).

We performed a multiple logistic regression to investigate the relationships between possible predictor variables and preparedness (Table 1). Seven variables were significantly associated with feeling better prepared for looking after patients with cancer; high conscientiousness; specific oncology teaching; days spent in a hospice; exposure to patients with cancer; relevant teaching; helpful communication skills training; and being able to identify role models. Four variables were not significantly associated with preparedness; attending a medical school with a problem-based learning course; possessing a prior degree; and feeling supported by senior colleagues and nursing staff.

In total, there were 16 questions about preparedness for aspects of cancer care in the questionnaire. A factor analysis of these 16 questions was performed to reduce the number of dependent variables and, thus, the chance of Type I error. An exploratory factor analysis with a Varimax rotation suggested four factors, together accounting for 56.8% of the variance. These were labelled ‘communication’, ‘recognising and diagnosing cancer’, ‘prescribing analgesia’, and ‘chemotherapy and radiotherapy knowledge’, according to the questions that loaded onto them. Respondents felt better prepared for ‘recognising and diagnosing cancer’ and ‘communication’ than for ‘prescribing analgesia’ or ‘chemotherapy and radiotherapy knowledge’. The respondent's preparedness for each factor was the mean of their preparedness for all the aspects that loaded onto that factor. For example, ‘prescribing analgesia’ was the mean of their preparedness for prescribing analgesia and for prescribing syringe drivers (Table 2).

The factor analysis identified four distinct aspects of cancer care (Table 2). We wanted to know whether specific elements of teaching may prepare students for specific aspects of cancer care, for example, hospice visits for prescribing analgesia. We performed a multiple linear regression to investigate relationships between elements of teaching and aspects of cancer care (Table 3). The resulting models demonstrated significant correlations between:

1. Specific oncology teaching, and feeling more prepared for ‘chemotherapy and radiotherapy knowledge’.
2. Spending time in a hospice, and feeling more prepared for ‘prescribing analgesia’, ‘communication’ and ‘recognising and diagnosing cancer’.
3. Meeting patients with cancer, and feeling more prepared for ‘recognising and diagnosing cancer’ and ‘communication’.
4. Meeting terminally ill patients, and feeling more prepared for ‘communication’, ‘prescribing analgesia’ and ‘chemotherapy and radiotherapy knowledge’.

The scale of these relationships is small. For example FY1 doctors who had seen 6–9 patients with cancer were on average 0.2 points more prepared for ‘recognising and diagnosing cancer’ than those who had met 2–5 patients. One interpretation of these results is that, with respect to preparedness for recognising and diagnosing cancer, exposure to patients with cancer is the most important element of oncology teaching.

## DISCUSSION

Oncology teaching and meeting patients with cancer are helpful in preparing students for looking after patients with cancer, but students' levels of exposure to patients with cancer are currently low. First foundation year doctors lack specialist knowledge about treatment of cancer including symptom control. First foundation year doctors and senior doctors agree that good communication is essential, but can be difficult in relation to patients with cancer.

Some of the reported relationships between aspects of undergraduate teaching and aspects of preparedness are small (see Table 1), while still being statistically significant (possibly through having a large sample). However, the validity of the conclusions is supported by the consistency between the quantitative results and the qualitative results.

The response rate in this study (43%) compares reasonably well to similar surveys of junior doctors in the UK, which have response rates from 9% through 33 to 66% (Goldacre *et al*, 2003; Roddy *et al*, 2004; Chard *et al*, 2006). The response rate of 66% was achieved by mailing questionnaires directly to doctors' home addresses as listed on the GMC register and sending four reminders, but this method can no longer be used because the release of doctors' addresses for research purposes is now prohibited by the Data Protection Act (Queen's Printer of Acts of Parliament, 1998).

Thirty-one percent of the FY1 doctors in this study recalled meeting fewer than 10 patients with cancer at medical school. This surprising finding may be explained by the way students categorise patients, that is, in terms of their physical signs rather than in terms of their diagnosis. For example, in the interviews, three FY1 doctors demonstrated evidence of depersonalisation, referring to patients with cancer as having ‘good signs’ or ‘brilliant livers’. An alternative explanation is that students were kept away from patients with cancer; however, this explanation was not backed up by the questionnaire findings. Students may be failing to register cancer patients as such, failing to see their medical school experiences with cancer patients as relevant with consequent detriment to their learning, or being kept away from patients with cancer without realising it.

There was an apparent contradiction in the results relating to chemotherapy and radiotherapy knowledge. First foundation year doctors felt unprepared in terms of chemotherapy and radiotherapy knowledge, and 75% said they would have preferred more teaching on this topic, but when we asked for the least important thing to learn about cancer the commonest answers were ‘details of chemotherapy’ and ‘details of radiotherapy’. Analysis of the qualitative data from the questionnaires suggested that respondents wanted to receive teaching about relevant aspects of chemotherapy and radiotherapy, for example side effects, rather than irrelevant details, for example schedules.

Previous studies have found that students from medical schools with problem-based learning feel better prepared than their colleagues from traditional style medical schools (Hill *et al*, 1998; Jones *et al*, 2002). We did not replicate this finding. This may be because the effect of problem-based learning upon preparedness for the FYI does not apply specifically to cancer, or because the effect of problem-based learning is mediated by one of the other variables we measured.

In 1992, the World Health Organisation (WHO) and the International Union Against Cancer (UICC) convened an international meeting to discuss undergraduate teaching. One of their recommendations was that students should spend a minimum of 2 weeks studying oncology (UICC, 1994). We have found that 39% of UK medical students leave medical school without having received specific oncology teaching. Furthermore, we found worryingly low levels of exposure to patients with cancer. If students are to learn about cancer, it is essential for them to meet patients with cancer at medical school. We recommend that oncologists maintain or increase their undergraduate teaching activities, and encourage greater involvement of patients in teaching. Moreover, oncology teaching should be made relevant to real life as a junior doctor, by placing emphasis on symptom control and avoiding unnecessary detail.

Specialist knowledge about cancer treatments changes rapidly. We have demonstrated that junior doctors are lacking certain specialist knowledge that is relevant to their practice. Oncologists could help to make specialist information more available to junior doctors by contributing to Internet-based reference material (e.g. www.cancerbackup.org.uk/).

We have found that communication skills are important for junior doctors looking after patients with cancer. This study was primarily about oncology teaching and did not look specifically at undergraduate training in communication skills. The relevance of the results to oncology teachers, however, is that junior doctors perceive the need for good communication where cancer patients are concerned. Oncologists may integrate communication skills into their teaching, for example, by acting as role models for good communication.

We believe that undergraduate curricula should place a greater emphasis on cancer. Oncology may become marginalised in systems-based curricula (Coles *et al*, 2003), and we recommend that medical educators flag up oncology as and when it arises in non-oncology-specific teaching. Improvements in doctors' skills should lead to improvements in the quality of care for our patients, because communication skills, management of symptoms, and feeling prepared for diagnosing cancer are important skills for doctors throughout their working lives, not just in their first postgraduate year.

Future studies about oncology teaching may ascertain the ideal duration and nature of oncology attachments, and may further quantify palliative care teaching. Future studies may also investigate objective (as well as subjective) measures of preparedness. For example, the FY1 doctors said they felt well prepared for breaking bad news, but their objective competence at doing so has not been studied.

## Figures and Tables

**Table 1:** Multivariate analysis showing the relationship between the questionnaire variables and PRHOs' preparedness for caring for patients with cancer (*n*=1814)

**Variable**	**Categories compared**	**Odds ratio (for feeling prepared) (95% CI)^a^**	***P*-value**
*Personality traits* b			
Agreeableness	5–15	0.95 (0.88–1.02)	0.16
Conscientiousness	5–15	1.10 (1.01–1.17)	0.02
Extraversion	5–15	1.02 (0.96–1.10)	0.50
Neuroticism	5–15	0.95 (0.90–1.00)	0.06
Openness	5–15	0.98 (0.93–1.03)	0.48
Doing an oncology attachment or special study module	Yes *vs* No	1.52 (1.14–2.04)	<0.01
Number of days spent visiting a hospice	0 *vs* <1 *vs* 1–2 *vs* 3–7	1.14 (1.02–1.29)	0.02
Number of patients with cancer seen at medical school	<2 *vs* 2–5 *vs* 6–9 *vs* >10	1.48 (1.22–1.79)	<0.001
Number of terminally ill patients seen at medical school	<2 *vs* 2–5 *vs* 6–9 *vs* >10	1.17 (1.03–1.34)	0.02
Having relevant teaching at medical school	Likert scale, 1–5	1.68 (1.47–1.91)	<0.001
Having helpful communication skills teaching at medical school	Likert scale, 1–5	1.27 (1.11–1.45)	<0.001
Having role models	Likert scale, 1–5	1.17 (1.05–1.30)	<0.01

a This column shows the odds of the FY1 doctors feeling prepared if they move one point higher up the scale upon which the variable was measured (e.g. if they see 6–9 patients with cancer instead of 2–5).

b NB all five personality traits are included in the model because they are measuring five aspects of one personality. Although only ‘conscientiousness’ is contributing to the model, overall personality is correlated with preparedness.

**Table 2:** The mean preparedness of the respondents for four major aspects of caring for patients with cancer: communication, recognition and diagnosis of cancer, prescribing analgesia, and chemotherapy and radiotherapy knowledge

**Factor (each factor represents one aspect of caring for patients with cancer)**	**Mean (95% CI) preparedness (scale of 1–10)**
Recognising and diagnosing cancer	7.1 (7.1–7.2)
Communication	6.9 (6.8–6.9)
Prescribing analgesia	5.4 (5.3–5.5)
Chemotherapy and radiotherapy knowledge	4.7 (4.7–4.8)

**Table 3:** Multiple regressions showing the relationships between different elements of training and preparedness for the four aspects of cancer care

	**Preparedness for different aspects of cancer care (scale 2–10)**			
**Aspects of undergraduate oncology teaching**	**Communication**	**Recognising and diagnosing**	**Prescribing analgesia**	**Chemotherapy and radiotherapy knowledge**
Did they receive specific oncology teaching? (Yes/No)	No significant contribution	No significant contribution	No significant contribution	*B*=0.61
	*P*=0.27	*P*=0.21	*P*=0.22	*P*<0.01
Number of patients with cancer seen (<2/2–5/6–9/>10)	*B*=0.10	*B*=0.21	No significant contribution	No significant contribution
	*P*=0.02	*P*<0.001	*P*=0.50	*P*=0.28
Number of days spent visiting a hospice	*B*=0.12	*B*=0.09	*B*=0.20	No significant contribution
(0/<1/1–2/3–7)	*P*<0.001	*P*<0.01	*P*<0.001	*P*=1.00
Number of terminally ill patients seen	*B*=0.28	No significant contribution	*B*=0.15	*B*=0.16
(<2/2–5/6–9/>10)	*P*<0.001	*P*=0.15	*P*<0.01	*P*<0.001

## References

[bib1] Australian Cancer Society (1999) Ideal Oncology Curriculum for Medical Schools. Sydney: Australia: Cancer Council Australia

[bib2] Bakemeier RF (1981) Cancer education objectives for medical schools. Med Pediatr Oncol 9: 585–633 7311939 10.1002/mpo.2950090604

[bib3] Barton MB, Bell P, Sabesan S, Koczwara B (2006) What should doctors know about cancer? Undergraduate medical education from a societal perspective. Lancet Oncol 7: 596–601 16814211 10.1016/S1470-2045(06)70760-4

[bib4] Barton MB, Simons RG (1999) A survey of cancer curricula in Australian and New Zealand medical schools in 1997. Oncology Education Committee of the Australian Cancer Society. Med J Aust 170: 225–227 10092922

[bib5] Barton MB, Tattersall MH, Butow PN, Crossing S, Jamrozik K, Jalaludin B, Atkinson CH, Miles SE (2003) Cancer knowledge and skills of interns in Australia and New Zealand in 2001: comparison with 1990, and between course types. Med J Aust 178: 285–289 12633488 10.5694/j.1326-5377.2003.tb05198.x

[bib6] Bender W, Haagedoorn EML, Oldhoff J (1993) Cancer Education in Europe according to Medical Faculty and Medical Students. the Netherlands: WHO Collaborating Centre for Cancer Education

[bib7] Boynton PM, Greenhalgh T (2004) Selecting, designing, and developing your questionnaire. BMJ 328: 1312–1315 15166072 10.1136/bmj.328.7451.1312 PMC420179

[bib8] Cellerino R, Graziano F, Piga A, Ghetti V (1993) The teaching of clinical oncology in Italian medical schools. A survey among teachers and students. Ann Oncol 4: 717–721 8280651 10.1093/oxfordjournals.annonc.a058654

[bib9] Chard D, Elsharkawy A, Newbery N (2006) Medical professionalism: the trainees' views. Clin Med 6: 68–71 10.7861/clinmedicine.6-1-68 PMC4954436 16521359

[bib10] Coles CR, Fleming WG, Golding LG (2003) Curricula for Cancer: A Practice Focused Approach. London: Cancer Research UK

[bib11] Costa PT (1992) Revised NEO Personality Inventory (NEO PI-R) and NEP Five-factor Inventory (NEO-FFI): Professional Manual. Odessa: Odessa, Fla

[bib12] Gaffan J, Dacre J, Jones A (2006) Educating undergraduate medical students about oncology: a literature review. J Clin Oncol 24: 1932–1939 16622269 10.1200/JCO.2005.02.6617

[bib13] Glaser B, Strauss AL (1967) The Discovery of Grounded Theory. Chicago: Adline

[bib14] Goldacre MJ, Lambert T, Evans J, Turner G (2003) Preregistration house officers' views on whether their experience at medical school prepared them well for their jobs: national questionnaire survey. BMJ 326: 1011–1012 12742922 10.1136/bmj.326.7397.1011 PMC154758

[bib15] Green J (1998) Commentary: grounded theory and the constant comparative method. BMJ 316: 1064–1065 9558994

[bib16] Greenhalgh T, Helman C, Chowdhury AM (1998) Health beliefs and folk models of diabetes in British Bangladeshis: a qualitative study. BMJ 316: 978–983 9550958 10.1136/bmj.316.7136.978 PMC28502

[bib17] Greenhalgh T, Seyan K, Boynton P (2004) ‘Not a university type’: focus group study of social class, ethnic, and sex differences in school pupils' perceptions about medical school. BMJ 328: 1541 15217871 10.1136/bmj.328.7455.1541 PMC437148

[bib18] Haagedoorn EML. Aspects of Cancer Education for Professionals (1985). the Netherlands: Gronigen University

[bib19] Hill J, Rolfe IE, Pearson SA, Heathcote A (1998) Do junior doctors feel they are prepared for hospital practice? A study of graduates from traditional and non-traditional medical schools. Med Educ 32: 19–24 9624395 10.1046/j.1365-2923.1998.00152.x

[bib20] International Union Against Cancer (UICC) (1994) Cancer Education for Undergraduate Medical Students: Curricula from around the world, Robinson E, Sherman CD, Love RR (eds). Undergraduate Education Around the World by E Robinson Geneva: UICC, pp 1–9

[bib21] Jones A, McArdle PJ, O'Neill PA (2002) Perceptions of how well graduates are prepared for the role of pre-registration house officer: a comparison of outcomes from a traditional and an integrated PBL curriculum. Med Educ 36: 16–25 11849520 10.1046/j.1365-2923.2002.01105.x

[bib22] Kalantar JS, Talley NJ (1999) The effects of lottery incentive and length of questionnaire on health survey response rates: a randomized study. J Clin Epidemiol 52: 1117–1122 10527007 10.1016/s0895-4356(99)00051-7

[bib23] Koczwara B, Barton MB (2006) The ideal oncology curriculum for medical students. J Clin Oncol 24: 5334 10.1200/JCO.2006.07.5465 17114670

[bib24] McManus IC, Keeling A, Paice E (2004) Stress, burnout and doctors' attitudes to work are determined by personality and learning style: a twelve year longitudinal study of UK medical graduates. BMC Med 2: 29 15317650 10.1186/1741-7015-2-29 PMC516448

[bib25] Office for National Statistics (2004) National Statistics, Health, Cancer, http://www.statistics.gov.uk/

[bib26] Pope C, Ziebland S, Mays N (2000) Qualitative research in health care. Analysing qualitative data. BMJ 320: 114–116 10625273 10.1136/bmj.320.7227.114 PMC1117368

[bib27] Queen's Printer of Acts of Parliament (1998) Data Protection Act 1998 Chapter 9

[bib28] Ramakrishnan S, Bolger JJ, Dunn KS, Neal FE, Hancock BW (1993) Undergraduate medical teaching in departments of oncology in the United Kingdom: a questionnaire survey. J Cancer Educ 8: 25–30 8489906 10.1080/08858199309528203

[bib29] Roddy E, Rubin P, Britton J (2004) A study of smoking and smoking cessation on the curricula of UK medical schools. Tob Control 13: 74–77 14985601 10.1136/tc.2003.004572 PMC1747835

[bib30] Rust J, Golombok S (1989) Constructing your own questionnaire. In Modern Psychometrics: The science of psychological assessment, pp 143–168. London: Routledge

[bib31] Silverman D (2005) Doing Qualitative Research. Sage: London

[bib32] Sofaer S (2002) Qualitative research methods. Int J Qual Health Care 14: 329–336 12201192 10.1093/intqhc/14.4.329

[bib33] Tattersall MH, Langlands AO, Smith W, Irwig L (1993) Undergraduate education about cancer. A survey of clinical oncologists and clinicians responsible for cancer teaching in Australian medical schools. Eur J Cancer 29A: 1639–1642 8217375 10.1016/0959-8049(93)90314-6

[bib34] The General Medical Council (1993) Tomorrow's Doctors. UK: The General Medical Council

[bib35] Wood DF (2003) Evaluating the outcomes of undergraduate medical education. Med Educ 37: 580–581 12834411 10.1046/j.1365-2923.2003.01564.x

